# Survival status and associated factors of death among cervical cancer patients attending at Tikur Anbesa Specialized Hospital, Addis Ababa, Ethiopia: a retrospective cohort study

**DOI:** 10.1186/s12885-019-6447-x

**Published:** 2019-12-16

**Authors:** Mulugeta Wassie, Zeleke Argaw, Yosief Tsige, Mesfin Abebe, Sezer Kisa

**Affiliations:** 1 School of Nursing, College of Medicine and Health Sciences, University of Gondar, Gondar, Ethiopia; 2 School of Nursing and Midwifery, College of Health Sciences, Addis Ababa University, Addis Ababa, Ethiopia; 3 Oslo University Collaboration Unit, Oslo, Norway

**Keywords:** Cervical cancer, Survival status, Tikur Anbesa Specialized Hospital, Ethiopia

## Abstract

**Background:**

Cervical cancer is a cancer of uterine cervix caused mostly by sexually-acquired infection called Human papillomavirus (HPV. In developing region of the globe, fewer than 50% of women with cervical malignancy survive more than 5 years. Therefore, the objective of this study was to assess survival status and associated factors of death among cervical cancer patients attending at Tikur Anbesa Specialized Hospital (TASH), Ethiopia.

**Methods:**

Facility based retrospective cohort study was conducted from March to April 2019 at Tikur Anbesa Specialized Hospital oncology center. Data was collected from patient’s chart using pre-tested and structured checklist prepared in English and analyzed using STATA14.2. Cox regression model was used to identify Variables that affected survival.

**Result:**

The overall survival rate was 38.62% at 5 years. There were a significance differences in survival experience between categories of stage of cervical cancer, age of patients, comorbidity, substance use, base line anemia and treatment modalities. Being stage IV [AHR = 11.76; 95% CI (4.02–34.4)],being advanced age [AHR = 5.99; 95% CI (2.1–17.08)], being comorbid [AHR = 1.58; 95%CI(1.14–2.19)], using substance [AHR = 1.56;95% CI(1.09–2.22)] and being anemic [AHR = 1.6;95% CI(1.11–2.36)] increased the risk of death.

**Conclusion:**

The overall survival rate was lower than high- and middle-income countries and Significant factors of death after diagnosis of cervical cancer were; advanced FIGO stage, base line anemia, comorbidity, substance use, advanced age and treatment modality. Author**s** recommend that it is better to expand cervical cancer early screening programs and treatment facilities, strengthen awareness in collaboration with public medias about cervical cancer prevention, screening and treatment options.

## Background

Cancer of uterine cervix was the fourth most commonly diagnosed malignancy of the women in 2012, approximately 527,600 incidences worldwide. Annually, more than two hundred seventy thousand women die with it in which greater than 85% of these mortalities are in less developed countries [1, 2].

Death of cervical cancer differed 18-fold within different regions of the globe, with rates lower than 2/100,000 in Western Asia, Western Europe and Australia while more than 20/100,000 in Melanesia (20.6), Middle Africa (22.2) and Eastern Africa (27.6). Similarly,in less-developed countries,less than 50% of women affected by cervical cancer survive longer than 5 years, while in economically advanced regions the 5 year survival is about 66% [1, 3]. For black cervical cancer patients, 5-year survival probability was lower than 25% and large variation in survival was observed even among developing countries due to variations in stages of presentation and accessibility to diagnostic and treatment services [4].

Care of cervical malignancy in Africa is also hampered by the absence of diagnostic and treatment facilities, healthcare infrastructures in addition to poor pathology services. In addition, there is a big brain drain of trained healthcare professionals in Africa that exacerbates the problem. Due to these reasons, cervical malignancy is projected to kill more than 474,000 women annually, in which 95% are anticipated to be in developing countries by 2030 [2, 5].

Approximately,35 cervical anaplasia cases were diagnosed for every 100,000 females, compared with only around 7 cases for every 100,000 women in North America and it is true in 2018 with an incidence of 6.6% death of 7.5% in Ethiopia [3, 6].

The occurrence of aggressive cervical carcinoma in cervical malignancy women was 15.6/1000 and the predisposing factors were revealed to be age more than 35,parity above six,significant low income or survival economy, mainly in individuals from the rural parts and prostitution [7].

Records showed that, of the approximately 22 million Ethiopian women with age above 15, roughly 7600 are identified with cancer of uterine cervix and about 6000 women die by the disease each year. These findings are perhaps significantly below the exact amount of cases, assumed the poor level of awareness, price, and inadequate access to screening services and absence of a countrywide cancer registry [8] .

Thirty-five point nine new cases of cervical cancer are diagnosed and 22.6 die from it per 100,000 women annually and many factors associated with are HPV, cultural factors like early marriage, poverty, coinfection and poor level of knowledge. There is an irregular and inconsistent cervical cancer screening and absence of standard policy and protocols leading women typically present for cancer care at late stage in the disease, where treatment is likely ineffective [9]. Due to such reasons, the emphasis of this study was to identify survival status and major factors associated to time to death, outcomes of cervical cancer and the degree to which these differences described by many factors.

## Methods

### Study design, period and area

Facility based retrospective cohort study was conducted at the oncology center of Tikur Anbesa Specialized Hospital (TASH) Addis Ababa, Ethiopia from March to April 2019. TASH is the biggest referral public hospital in Ethiopia. It is the training center of health professionals including undergraduate and postgraduate students. The hospital is staffed by many health professionals from several disciplines. It has total beds of 800 and the beds reserved for cancer care at oncology center are 20. The oncology center of TASH is providing chemotherapy, radiation therapy, complain therapy and other supportive and palliative cares. It is the major area for cancer registry, early detection, prevention, standard treatment and palliative care in Addis Ababa and it is the only cancer center in the Ethiopia.

### Population

All medical records of women diagnosed with cervical cancer in Tikur Anbesa Specialized Hospital attending the oncology center were source populations and all medical records of cervical cancer patients who attended the oncology center of TASH from January 12,014 to December 31,2016 were study populations. Study populations fulfilling inclusion criteria were considered as sample populations. All cervical cancer patients diagnosed and treated at TASH from January 12,014 to December 31,2016 was the inclusion criteria of the study and patients whose medical charts were incomplete, medical charts not found and patients who had been previous diagnosis of cervical cancer and registered during the data collection times were excluded.

### Sample size determination, sampling procedure and study variables

All cervical cancer patients’ medical records diagnosed and treated at TASH oncology center from January 12,014 to December 31,2016 was the total sample size. Census sampling procedure was used and the procedure was as follows: at the beginning, profiles of all cervical cancer women on follow up between January 12,014 to December 31,2016 in the TASH was assessed and 1227 cervical cancer patients’ charts were found. Six hundred thirty-four (634) charts which fulfilled the inclusion criteria were identified by data collectors from list of cervical cancer charts that are on cancer care follow up at TASH oncology center. Finally, data was collected from 634 patients’ medical charts. Time to death was the outcome variable and sociodemographic and individual level factors (marital status, residential address, age at diagnosis, substance use, number of children, region, occupation, religion), pathological and clinical factors (stage at presentation, histology type, bassline anemia, comorbidity, types of comorbidity) and Treatment related factors (Chemotherapy, Radiation,Surgery,Aim of radiotherapy,Combination of treatments modalities) were independent variables.

### Operational definition

Event: Death of cervical cancer patients was an event of interest for this research.

Censored: Patients who loss follows up, not die up to the study period and those transfer to different care unit during the study.

Comorbidity: The presence of any conditions (mentioned in the Carlson comorbidity Index [10]) other than cervical cancer at diagnosis which was designated as “yes” in the checklist.

Substance use: Patients who used one, two or all of the three substances (cigarette, chat and alcohol) [11].

Anemia: patients hemoglobin levels below 12.0 g/dl was classified as anemic [12].

Entry date and closing date to follow-up: Entry date was the first date of clear diagnosis of cervical cancer (January1^st^ 2014 to December31st, 2016). Closing date was the ending date to follow up (December31^st^, 2018).

Survival status: Survival status was defined as the outcome of patients which was classified into censored or death from patient’s clinical data file from scheduled or unscheduled visits.

Time to death: Time to death was calculated at the time between the date of clear diagnosis of cervical cancer to the date of death (in month).

Stage at diagnosis: The revised FIGO staging for carcinoma of the vulva, cervix, and endometrium was used in this study [13].

### Data collection instrument, data collection procedures and pre-test

Data was collected from patients’ charts using pre-tested and structured checklist prepared in English. The checklist contains three parts: (1) sociodemographic and individual level factors, (2) Pathological and clinical factors and (3) Treatment related factors. Two supervisors having second degree in oncology nursing and three data collectors having first degree in nursing were involved in the data collection process. Next to recruitment of information collectors and monitors, one-day intensive training was given. Pretest was conducted by considering 5% of the total sample size to test its consistency with actual data collection in the charts recorded during the study period. After pretest, the checklists were assessed for its clarity, length and completeness and the necessary corrections were done accordingly.

### Data quality assurance procedures

The quality of data was assured by proper designing and pre-testing of the checklists and by giving training for the data collectors and supervisors before the actual data collection. Appropriate modifications were made after viewing the pre-test result and overall supervision was made by the principal investigator. Every day after data collection, checklists were reviewed and checked for completeness, accuracy and clarity by the supervisors and principal investigator and the necessary correction was given to data collectors in the following morning.

### Data processing and analysis

Data were coded and then entered, edited and cleaned using EPI-data 3.1 and exported to STATA14.2 statistical software for analysis. Frequencies, proportions and descriptive statistics was used to explain the study population in relation to relevant variables and was presented using tables and graphs.

Kaplan Meier analyses with life table were used to identify the overall survival rates and median survival time. Differences in survival among different variables were compared using the log-rank test. Before running the Cox regression model, assumption of proportional-hazard was performed. Cox-proportional hazard model assumption was checked using Schoenfeld residual test and variables having *P*-value > 0.05 were considered as fulfilling the assumption.

Variables with a significance level below 0.2 in the bivariable Cox regression model were included in a multivariable Cox regression model analysis. Variables in multivariable Cox model with a *p*-value < 0.05 were considered to have actual interference with the survival of the patients with 95%confidence interval. The datasets used and/or analyzed during the current study are available from the corresponding author on reasonable request.

### Ethical clearance

Ethical approval for this study was obtained from the Institutional Review Boards of school of nursing and midwifery, Addis Ababa University.

## Result

### Socio-demographic characteristics of the study participants

Between January 1^st^2014 to December 31^st^ 2016, 1227 cervical cancer patients were enrolled to Tikur Anbesa Specialized Hospital oncology center from which 634 were eligible for this study. The median age of study participants was 50 months with SD ± 11.72. The minimum and maximum ages of patients were15 and 87 years respectively. Fifty six percent (56%) of participants were urban dwellers and 32% came from Oromia region. More than half (58.2%) of the study participants were Orthodox religion followers and 42.4% were house wives. Nearly one sixth (16.7%) use substances, about two third (64.2%) were married and 42% have more than three children (Table 1).

**Table 1:** Socio demographic characteristics of cervical cancer patients in Tikur Anbesa Specialized Hospital oncology center, Ethiopia (*n* = 634)

Covariates	Code	Status at last contact		Total No (%)
		Censored No (%)	Death No (%)	
Age	< 30	55 (93.2)	4 (6.8)	59 (9.3)
	30–39	100 (84.0)	19 (16.0)	119 (18.8)
	40–49	118 (72.0)	46 (28.0)	164 (25.9)
	50–59	101 (65.2)	54 (34.8)	155 (24.4)
	≥60	74 (54.0)	63 (46.0)	137 (21.6)
Residence	Urban	253 (71.3)	102 (28.7)	355 (56)
	Rural	195 (69.9)	84 (30.1)	279 (44)
Region	Amhara	104 (64.6)	57 (35.4)	161 (25.4)
	Oromia	146 (71.9)	57 (28.1)	203 (32)
	Tigray	14 (60.9)	9 (39.1)	23 (3.6%)
	SNNP	41 (68.3)	19 (31.7)	60 (9.5)
	Addis Ababa	127 (76.0)	40 (24.0)	167 (26.3)
	Others	16 (80.0)	4 (20.0)	20 (3.2)
Religion	Orthodox	257 (69.6)	112 (30.4)	369 (58.2)
	Muslim	94 (70.7)	39 (29.3)	133 (21)
	Protestant	87 (73.1)	32 (26.9)	119 (18.8)
	Others	10 (76.9)	3 (23.1)	13 (2.1)
Occupation	Gov’t employee	63 (74.1)	22 (25.9)	85 (13.4)
	Merchant	44 (60.3)	29 (39.7)	73 (11.5)
	Farmer	77 (65.8)	40 (34.2)	117 (18.5)
	house wife	197 (73.2)	72 (26.8)	269 (42.4)
	Others	67 (74.4)	23 (25.6)	90 (14.2)
Substance use	User	51 (48.1)	55 (51.9)	106 (16.7)
	None user	397 (75.2)	131 (24.8)	528 (83.3)
Children	no child	18 (90)	2 (10)	20 (3.2)
	One	27 (65.9)	14 (34.1)	41 (6.5)
	Two	64 (69.6)	28 (30.4)	92 (14.5)
	Three	138 (64.2)	77 (35.8)	215 (33.9)
	more than 3	201 (75.6)	65 (24.4)	266 (42)
Marital status	Married	287 (70.5)	120 (29.5)	407 (64.2)
	Single	22 (71)	9 (29)	31 (4.9)
	Widowed	65 (66.3)	33 (33.7)	98 (15.5)
	Divorced	74 (75.5)	24 (24.5)	98 (15.5)

### Clinical, histopathological and treatment characteristics

About two third (65.1%) of cervical cancer patients presented at advanced stages (III&IV), 574 (90.5%) had squamous cell carcinoma, nearly half (50.9%) were anemic during presentation. About one third (33.4%) had comorbidity and from those who had comorbidity, more than half (54.7%) were HIV positives. Nearly half (50.3%) took radiation treatment only. Three hundred ninety (61.5%) of patients took palliative radiotherapy (Tables 2 and 3)

**Table 2:** Clinical and histopathological characteristics of cervical cancer patients in Tikur Anbesa Specialized Hospital oncology center, Ethiopia (*n* = 634)

Covariates	Code	Status at last contact		Total No (%)
		Censored No (%)	Death No (%)	
FIGO stage	Stage I	59 (93.7)	4 (6.3)	63 (9.9)
	Stage II	132 (83.5)	26 (16.5)	158 (24.9)
	Stage III	184 (72.2)	71 (27.8)	255 (40.2)
	Stage IV	73 (46.2)	85 (53.8)	158 (24.9)
Histopathology	Squamous cell	406 (70.7)	168 (29.3)	574 (90.5)
	Adenocarcinoma	42 (70.0)	18 (30.0)	60 (9.5)
Anemic status	Yes	184 (57.0)	139 (43.0)	323 (50.9)
	No	264 (84.9)	47 (15.1)	311 (49.1)
Comorbidity	Yes	117 (55.2)	95 (44.8)	214 (33.4)
	No	331 (78.4)	91 (21.6)	422 (66.6)
Types of comorbidity	HIV	56 (47.5)	62 (52.5)	118 (55)
	Hypertension	29 (59.2)	20 (40.8)	49 (23)
	Others	33 (70.2)	14 (29.8)	47 (22)

**Table 3:** Treatment related characteristics of cervical cancer patients in Tikur Anbesa Specialized Hospital oncology center, Ethiopia (*n* = 634)

Covariate	Code	Status at last contact		Total No (%)
		Censored No (%)	Death No (%)	
Treatment initiated	Surgery and chemo	7 (77.8)	2 (22.2)	9 (1.4)
	Chemotherapy only	13 (81.3)	3 (18.8)	16 (2.5)
	Radiotherapy only	228 (71.5)	91 (28.5)	319 (50.3)
	Chemo radiation only	142 (66.7)	71 (33.3)	213 (33.6)
	surgery & radiation only	51 (78.5)	14 (21.5)	65 (10.3)
	Surgery+chemo+radation	7 (58.3)	5 (41.7)	12 (1.9)
Chemotherapy cycles	No chemo	235 (70.6)	98 (29.4)	333 (52.5)
	First cycle	69 (75)	23 (25.0)	92 (14.5)
	Second cycle	59 (68.6)	27 (31.4)	86 (13.6)
	Third cycle	34 (77.3)	10 (22.7)	44 (6.9)
	Fourth cycle	24 (61.5)	15 (38.5)	39 (6.2)
	Others	27 (67.5)	13 (32.5)	40 (6.3)
Aim of radiation treatment	Radical	178 (82.8)	37 (17.2)	215 (33.9)
	no radiation	26 (89.7)	3 (10.3)	29 (4.6)
	Palliative	244 (62.6)	146 (37.4)	390 (61.5)

### Overall survival of cervical cancer patients during the follow-up time

**Fig. 1:**
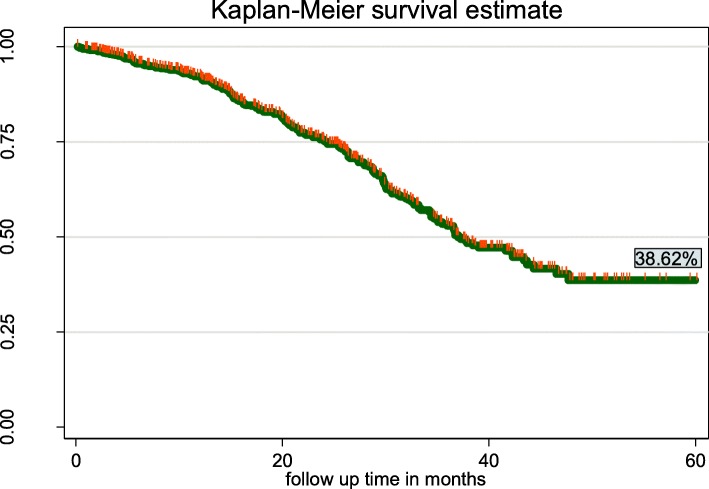
Overall Kaplan-Meier estimation of survival of cervical cancer patients at Tikur Anbesa Specialized Hospital oncology center, Ethiopia (*n* = 634)

In this study, 634 cervical cancer patients were followed for 60 months (5 years). The median survival of this cohort was 37.03 months. The overall estimated survival rate of cervical cancer patients was 38.62% at 60 months of follow up. The estimated cumulative survival was 92.11,75.29%, 52.92, 38.62 and 38.62% at 12, 24, 36, 48 and 60 months respectively as shown in the following Kaplan Meir survival curve (Fig. 1).

### Survival experience among different groups of cervical cancer patients

**Fig. 2:**
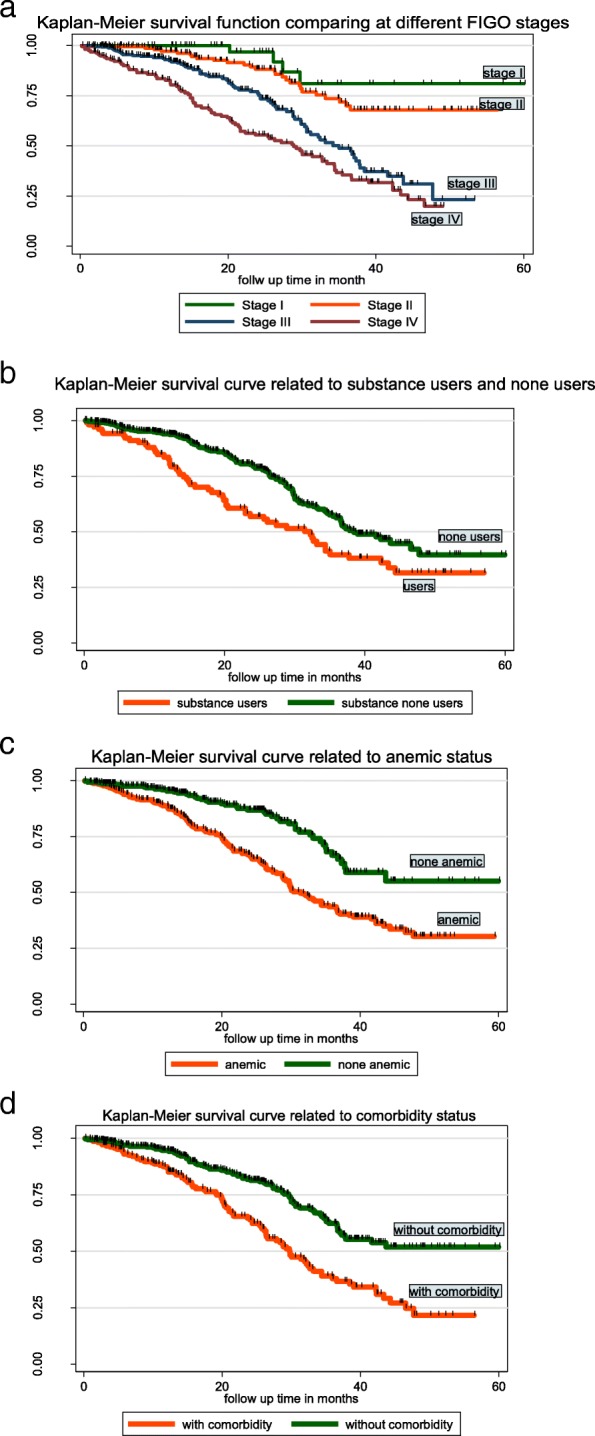
**a** The Kaplan-Meier survival curves compare survival time of cervical cancer patients with different FIGO stages in Tikur Anbesa Specialized Hospital oncology center, Ethiopia (*n* = 634). **b** The Kaplan-Meier survival curves compare survival time of cervical cancer patients related to substance users and none users in Tikur Anbesa Specialized Hospital oncology center, Ethiopia (*n* = 634). **c** The Kaplan-Meier survival curves comparing survival time of cervical cancer patients related to anemic status in Tikur Anbesa Specialized Hospital oncology center, Ethiopia (*n* = 634). **d** The Kaplan-Meier survival curves comparing survival time of cervical cancer patients related comorbidity status at Tikur Anbesa Specialized Hospital oncology center, Ethiopia (*n* = 634)

Statistical difference in survival time between different categories of covariates was tested using Log-rank test. It was found that there is a significant difference in survival experience between categories of stage of cervical cancer, substance use, age of patients, comorbidity, anemic status and aim of radiation therapy (RT) at *p*-value < 0.05 (Fig. 2a, b, c and d).

The median survival time for those who had FIGO stage I, II or III at baseline had a longer survival time than those in stage IV (28.87 months) (95% CI:21.6–34.37). The median survival time for those who are none substance users had a longer survival time (38.46 months; 95%CI: 35.93–47.7) than those who are substance users (32.2 months) (95% CI:20.5–37.77). For cervical cancer cases diagnosed at early stage (I&II), cumulative survival rate was 81.04 and 67.94% respectively and those cases diagnosed at advanced stage (III&IV) was 23.33 and 20.03% respectively (Table 4).

**Table 4:** Median survival time, cumulative survival probability, significance and log rank test of cervical cancer patients at Tikur Anbesa Specialized Hospital oncology center, Ethiopia (*n* = 634)

Covariates	Median survival time, In month (95% CL)	Overall 5-year Survival (%)	Log rank test	*p*–value
Age			39.87	< 0.001
< 30	^a^	81.45		
30–39	^a^	56.51		
40–49	39 (31.93–43.3)	41.42		
50–59	32.2 (27.37–38.46)	21.29		
≥ 60	30.37 (26.07–35)	22.30		
Substance			10.63	0.0011
User	32.2 (20.5–37.77)	31.57		
None User	38.46 (35.93–47.7)	39.62		
FIGO Stage			56.68	< 0.001
I	^a^	81.04		
II	^a^	67.94		
III	35 (30.37–37.87)	23.33		
IV	28.87 (21.6–34.37)	20.03		
Anemic Status			27.05	< 0.001
Yes	31.4 (28.87–36.53)	30.26		
No	^a^	55.05		
Comorbidity			27.78	< 0.001
Yes	29.8 (26.33–33.17)	21.55		
No		51.80		
Aim RT			21.05	< 0.001
Radical	46.53 (34.4–51.52)	46.16		
Palliative	34.67 (30.03–37.3)	32.57		
No RT	^a^	82.8		

^a^values that hadn’t median survival from the specific categorical variable

### Factors associated with survival time among cervical cancer patients

The independent variables were analyzed individually with the outcome variable and those variables fitted at *p*-value < 0.2 were included in the multivariate Cox regression model.

The proportionality of hazard assumption was checked using the Schoenfeld residual test and it satisfied at p-value > 0.05 of independent factors.

The result of multivariable analysis revealed that women with FIGO stages IV and stage III were11.76 times [AHR = 11.76,95% CI (CI:4.02–34.4)]and 7.4 times [AHR = 7.4,95% CI(2.55–21.7)] respectively at high risk to die as compared to those with early FIGO stage I as a reference. Those women with advanced age (≥60) were 5.99 times [AHR = 5.99,95% CI (2.1–17.08)] at high risk to die than those with early age (≤30).

Patients who used substance were 1.56 times [AHR = 1.56,95% (CI: 1.09–2.22)] at high risk to die than those who were none substance users. Similarly, cervical cancer patients who have comorbidity were 1.58 times [AHR = 1.58,95% CI (1.14–2.19)] at higher hazard to develop the event than those who haven’t comorbidity and patients who were anemic were 1.6 times [AHR = 1.6,95% CI (1.11–2.36)] at high risk to die than with those without anemia. Patients who were treated with surgery and radiation were 0.70 times [AHR = 0.30,95% CI (0.10–0.93)] at lower hazard to die than those patients treated with combination of chemotherapy, surgery and radiation therapy (Table 5).

**Table 5:** Results of the bivariable and multivariable cox regression analysis of cervical cancer patients at Tikur Anbesa Specialized Hospital oncology center, Ethiopia (*n* = 634)

Covariates	Bivariable		Multivariable	
	*p*-value	CHR (95% CI)	*p* -value	AHR (95% CI)
Age				
< 30		1		1
30–39	0.095^*^	2.5(0.85–7.4)	0.317	1.76(0.58–5.28)
40–49	0.003*	4.9(1.7–13.3)	0.103	2.4(0.84–6.94)
50–59	< 0.001*	6.6(2.39–18.4)	0.002**	5.4(1.91–15.31)
≥ 60	< 0.001*	7.97(2.89–21.97)	0.001**	5.99(2.1–17.08)
Occupation				
Government employee		1		1
Merchant	0.024*	1.9(1.09–3.31)	0.058	1.74(0.98–3.11)
Farmer	0.154^*^	1.5(0.86–2.46)	0.189	1.4(0.83–2.5)
House wife	0.664	1.1(0.69–1.79)	0.677	1.11(0.66–1.87)
Others	0.926	1.03(0.57–1.84)	0.381	0.75(0.40–1.42)
Substance use				
User	0.001*	1.7(1.23–2.224)	0.013**	1.56(1.09–2.22)
None user		1		1
No. of children				
No child		1		1
One	0.026*	5.4(1.23–23.78)	0.119	6.25(0.35–28.9)
Two	0.081*	3.6(0.85–15.08)	0.173	3.9(0.88–16.66)
Three	0.055*	3.9(0.97–16.11)	0.111	3.24(0.76–13.8)
More than three	0.040*	4.4(1.07–17.9)	0.098	3.43(0.79–14.8)
FIGO stage				
Stage I		1		1
Stage II	0.160*	2.13(0.74–6.1)	0.007**	4.6(1.53–13.94)
Stage III	0.001*	5.24(1.91–14.36)	< 0.001**	7.4(2.55–21.7)
Stage IV	< 0.001*	8.14(2.98–22.18)	< 0.001**	11.76(4.02–34.4)
Anemic status				
Yes	< 0.001*	2.35(1.68–3.27)	0.011**	1.6(1.11–2.36)
No		1		1
Comorbidity				
Yes	< 0.001*	2.13(1.59–2.83)	0.006**	1.58(1.14–2.19)
No		1		1
Treatment				
Surgery &chemo	0.261	0.39(0.07–2.01)	0.792	0.78(0.13–4.8)
Chemotherapy	0.528	0.63(0.15–2.64)	0.942	1.06(0.20–5.62)
Radiotherapy	0.318	0.63(0.26–1.56)	0.271	0.49(0.13–1.74)
Chemo radiation	0.505	0.73(0.29–1.82)	0.092	0.42(0.16–1.15)
Surgery& radiation	0.064*	0.37(0.14–1.06)	0.037**	0.30(0.10–0.93)
Combination of the three modalities		1		1
Others		1		1
Aim of Radiation				
Radical		1	1	
No RT	0.357	0.58(0.177–1.87)	0.477	0.61(0.15–2.41)
Palliative	< 0.001*	2.1(1.45–2.99)	0.246	1.26(0.85–1.88)

*Indicates the variables significantly associated with the outcome variable in the bivariable analysis at 푃 < 0.2), while ** indicates the variables significantly associated with the outcome variable at 푃 < 0.05 in multivariable analysis with 95% confidence level

## Discussion

The current study demonstrated that, about 38.6% cervical cancer patients could survive at least 5 years after diagnosis of cervical cancer. This is significantly lower than the researches conducted in Brazil which were 84% [14], India 62% [15], Malaysia 71.1% [16],China 66.61% [17],Northwest Russia 60.0% [18],Mysuru (India) 48.1% [19], but in line with the study conducted in North East India 40.7% [20].

About 92,75%, 53, 39 and 38.6% of women with cervical cancer could survive at least one, two, three, four and 5 years respectively in the current study. This is lower than the study conducted in Malaysia which was 94.1, 79.3 and 71.1% at one, three and 5 years respectively [17].

This discrepancy might be due to differences in sample size, study period as there could be changes in treatment modality, health care policy related to cervical cancer, treatment modalities and availability of health-related infrastructures that support early detection and treatment of the cervical cancer.

Nearly 88% of cervical cancer patients who took palliative radiation therapy and 97% of those who took radical radiation therapy survived at least 1 year from this research.

The current result is in agreement with study conducted on the effect of adherence of radiation therapy for one-year survival in Ethiopia which was 96% for radical therapy and 87% for those who took none radical radiation therapy [21]. This may be due to the similarity of study setting, treatment modalities and roughly the same sample sizes of study participants.

Fifty percent chance of surviving of an individual woman with cervical cancer in this study was about 37 months. This finding is higher than a study conducted in Kenya which was 15 months [22], but lower than Malesia 65.8 months [17] and North East India 44 months [20].

The above median survival time dissimilarities may be due to study period variation, the stage and overall condition of patients during presentation, variations of waiting time for treatment after confirmed cervical cancer diagnosis and cancer care policy differences. However, the median survival time result of this study is in agreement with the study conducted in Ethiopia,that was 38 months [23]. This similarity will be due to the similarity of study settings, cancer care policy of the country Ethiopia which is unchanged till, the stage of the disease at presentation, and overall care of cervical cancer patients in TASH oncology center.

Patients with advanced age (≥60 and 50–59) during presentation were about 6 and 5.4 times at high risk to die than those with early age (≤30) as a reference respectively.

This finding is supported by the studies conducted in Danish (HR, 1.60; 1.29–1.98) and Mysuru (HR = 2.17, *p* = 0.05) [19, 24]. This agreement will be due to the fact that the death of cervical cancer increases as the age of women with this disease increases [25].

Stage at presentation was the contributing factor for low survival of cervical cancer patients in this study. Women with FIGO stage IV and III were about 12 and 7.4 times at high risk to die as compared to those with early clinical stage I respectively. This finding is in line with the studies conducted at north west Russia (HR = 3.8, 95% CI 2.5 to 5.8) [18], Nigeria (HR = 3.3, 95% CI: 1.2–8.9) [26], Ethiopia (HR = 2.60 (95%CI: 1.67–4.04) [23] and North East India (HR =1.8,95% CI:1.2 to 2.7) [20]. The similarity of these findings may be due to the reason that as the cancer stage increases, metastasis will also increase, which leads to compromising of patient’s immunity to resist other infections, difficulty of treating such complicated metastasis cases and these factors in aggregate increases the probability of dying [13].

Patients who were anemic in this study were 1.6 times at high risk to die than with those patients without anemia. This result is supported by the studies conducted at Nigeria (HR = 3.0, 95% CI: 1.4, 6.4) [26] and Ethiopia (HR = 1.65 (95%CI: 1.24–2.20) [23]. This agreement could be due to the fact that low level of hemoglobin in the blood can lead to oxygen starvation of both cancerous and normal cells that consequently results unprogrammed cell death. Cervical cancer patients who had comorbidity were 1.58 times at high risk to develop the event than those who haven’t comorbidity in this study. This is in agreement with the studies conducted in Australian [HR = 4.6,95% (CI: 3.54–6.03)] [27] and Ethiopia [HR = 2.02, 95% CI: 1.01–4.05) [23]. The study on HIV infection and survival among women with cervical cancer in Botswana indicated that HIV infection significantly increased the risk for death among all women with cervical cancer [HR = 1.95;95%CI(1.20 to 3.17)] [28] and this also strengthen the current study result since prevalence of HIV infection from comorbidity lists was the highest. The rational of these similarities may be due to the fact of Carlson comorbidity index which states as the presence of comorbidity shortens the life based on the severity of each comorbidity [10].

Another significant finding in this research that lowered survival probability was substance use (Chat chewing, alcohol drinking and cigarette smoking). Patients who used substance in this study were 1.56 times at higher risk to die than those who don’t used substances. The result of this study may be due to substances which were used by the patients weekend their immunity which leads to be attached by other infectious diseases that lower survival.

### Limitations and strength of the study

This study has some limitations. First, cause specific (relative) survival was not determined due to lack of data on specific cause of death, this may over estimate cervical cancer related mortality rate Moreover, the data were collected on patients registered from January one 2014 to December 31 2016 that may not reflect current utilization of advanced treatment modalities, which could affect the opportunity to improve survival rate.

As a strength, the study left two-years for follow up period in addition to 3 years entry periods which probably increased the number of events and data collected by nurses who were trained on cancer care that could increase the quality of the data.

## Conclusions

The five-year overall probability of survival rate among cervical cancer patient was 38.62%, which is lower when compared with those of high- and middle-income countries. We recommend that it is better to expand cervical cancer early screening programs and cervical cancer treatment facilities and better to create awareness in collaboration with public medias about cervical cancer prevention, screening, treatment options and early symptoms.

Furthermore, prospective follow up study could be conducted by incorporating important predictors of mortality like financial problems, treatment adherence, societal and health system related factors.

## Data Availability

Data will be available upon request from the corresponding author.

## Abbreviations

AHR: Adjusted Hazard Ratio
AIDS: Acquired Immune Deficiency Syndrome
CC: Cervical Cancer
CHR: Crude Hazard Ratio
CI: Confidence Interval
ETB: Ethiopian Birr
FIGO: International Federation of Gynecology and Obstetrics
HPV: Human Papillomavirus
HR: Hazards Ratio
ICC: Invasive Cervical Cancer
RT: Radiation Therapy
TASH: Tikur Anbesa Specialized Hospital

## References

[1] Alves C, Alves L, Lunet N (2007). Epidemiology of cervical cancer. Cancer.

[2] Denny L, Anorlu R (2012). Cervical cancer in Africa. Cancer Epidemiol Prev Biomarkers.

[3] Cecilia NC, Rosliza A, Suriani I (2017). Global burden of cervical Cancer. Int J Public Health Clin Sci.

[4] Sankaranarayanan R (2006). Overview of cervical cancer in the developing world. Int J Gynecol Obstet.

[5] Wittet S, Goltz S, Cody A (2015). Progress in cervical cancer prevention: the CCA report card 2015.

[6] Bray F (2018). Global cancer statistics 2018: GLOBOCAN estimates of incidence and mortality worldwide for 36 cancers in 185 countries. CA Cancer J Clin.

[7] Pelzer A (1992). A study of cervical cancer in Ethiopian women. Cytopathology.

[8] Woldeamanuel YW, Girma B, Teklu AM (2013). Cancer in Ethiopia. Lancet Oncol.

[9] Asseffa NA (2017). Cervical Cancer: Ethiopia’s Outlook. J Gynecol Womens Health.

[10] Charlson M, Foley W (2018). Charlson comorbidity index: chart review version.

[11] World Health Organization. Promoting mental health: concepts, emerging evidence, practice: a report of the World Health Organization: Department of Mental Health and Substance Abuse in collaboration with the Victorian Health Promotion Foundation and the University of Melbourne. Geneva: World Health Organization; 2005.

[12] CSA (2016). Ethiopia demographic and health survey 2016: key indicators report.

[13] Pecorelli S (2009). Revised FIGO staging for carcinoma of the vulva, cervix, and endometrium. Int J Gynecol Obstet.

[14] Carneiro SR (2017). Five-year survival and associated factors in women treated for cervical cancer at a reference hospital in the Brazilian Amazon. PLoS One.

[15] Jayant K (2016). Improved survival of cervical Cancer patients in a screened population in rural India. Asian Pac J Cancer Prev.

[16] Muhamad NA (2015). Survival rates of cervical cancer patients in Malaysia. Asian Pac J Cancer Prev.

[17] Chen J-G (2018). Cancer survival in patients from a hospital-based cancer registry, China. J Cancer.

[18] Grjibovski AM (2018). Incidence, mortality and determinants of survival from cervical cancer in Northwest Russia: a registry-based cohort study. Int Health.

[19] Vishma B (2017). Survival and prognostic factors for cervical cancer: a hospital based study in Mysuru, India. Int J Commun Med Public Health.

[20] Kataki AC (2018). A survival study of uterine cervical patients in the north East India: hospital-cancer registry-based analysis. J Cancer Res Ther.

[21] Moelle U (2018). Cervical Cancer in Ethiopia: The Effect of Adherence to Radiotherapy on Survival. Oncologist.

[22] Maranga IO (2013). Analysis of factors contributing to the low survival of cervical cancer patients undergoing radiotherapy in Kenya. PLoS One.

[23] Gizaw M (2017). Cervical cancer patients presentation and survival in the only oncology referral hospital, Ethiopia: a retrospective cohort study. Infect Agents Cancer.

[24] Ibfelt E (2013). Socioeconomic position and survival after cervical cancer: influence of cancer stage, comorbidity and smoking among Danish women diagnosed between 2005 and 2010. British J Cancer.

[25] Ghim S, Basu PS, Jenson A (2002). Cervical cancer: etiology, pathogenesis, treatment, and future vaccines. Asian Pac J Cancer Prev.

[26] Musa J (2016). Cervical cancer survival in a resource-limited setting-north Central Nigeria. Infect Agents Cancer.

[27] Diaz A (2018). Comorbidity and cervical cancer survival of indigenous and non-indigenous Australian women: a semi-national registry-based cohort study (2003-2012). PLoS One.

[28] Dryden-Peterson S (2016). HIV infection and survival among women with cervical cancer. J Clin Oncol.

